# Materiovigilance in Perspective: Understanding Its Concept and Practice in the Global Healthcare System

**DOI:** 10.1007/s43441-023-00514-4

**Published:** 2023-04-27

**Authors:** Binaya Sapkota, Subish Palaian, Sunil Shrestha, Mohamed Izham Mohamed Ibrahim

**Affiliations:** 1 Faculty of Health Sciences, Nobel College, Sinamangal, Kathmandu, Nepal; 2 Jeffrey Sachs Center On Sustainable Development, Sunway University, Sunway, Selangor Malaysia; 3 Department of Clinical Sciences, College of Pharmacy and Health Sciences, Ajman University, Ajman, United Arab Emirates; 4 School of Pharmacy, Monash University Malaysia, Jalan Lagoon Selatan, Bandar Sunway, 47500 Sunway, Selangor Malaysia; 5 College of Pharmacy, QU Health, Qatar University, Doha, Qatar

**Keywords:** Device recall, Materiovigilance, Medical devices, Medical devices associated adverse events (MDAEs), Pharmacovigilance, Regulation, Reporting guidelines

## Abstract

Materiovigilance (Mv) has the same purpose and approach in ensuring patient safety as pharmacovigilance but deals with medical devices associated with adverse events (MDAEs) and their monitoring. Mv has been instrumental in recalling many defective or malfunctioning devices based on their safety data. All MDAEs, such as critical or non-critical, known, or unknown, those with inadequate or incomplete specifications, and frequent or rare events should be reported and evaluated. Mv helps to improve medical devices’ design and efficiency profile and avoid device-related complications and associated failures. It alerts consumers and health professionals regarding counterfeit or substandard devices. Common events reported through Mv are device breakage and malfunction, entry- and exit-site infections, organ perforations or injuries, need for surgery and even death, and life cycle assessment of devices. Health authorities globally have developed reporting frameworks with timeframes for MDAEs, such as MedWatch in the USA, MedSafe in New Zealand, and others. Health professionals and consumers need to be made aware of the significance of Mv in ensuring the safe use of medical devices and getting familiar with the reporting procedures and action plans in case of a device-induced adverse event.

## Introduction

Technological advances have significantly influenced healthcare practices, resulting in improved healthcare outcomes. Many technological advancements in recent times, such as drug device-combined modalities, automation and robotics, sophisticated devices, and others, caused a rapid upsurge in the applications of medical devices in healthcare facilities (HCFs) [1]. Medical devices are widely used to diagnose, prevent, and treat different diseases [2] and range from simple meter dose inhalers to complicated operation theater and radiology devices. The global demand for medical devices has risen exponentially and reached 380 billion USD in 2016 from 260 billion USD in 2006, with the burgeoning incidence of stroke, obesity, diabetes, cancer, and many other chronic diseases [1, 3]. Hence, with each new product being launched into the market, practitioners, policymakers, regulators, and patients must become aware of their safety, effectiveness, quality assurance, and other essential parameters, which long-term pharmacovigilance (Pv) activities can explore. A similar concept and approach of Pv have been incorporated in medical devices, termed materiovigilance (Mv).

While the use of medical devices is increasing, the concerns regarding their safety are also rising. Like medicines, the post-marketing data of the applications and effects of medical devices on healthcare are also vital sources of evidence to ensure their safety and performance characteristics [4]. It is usual to report adverse events during phases mandatorily I to III of clinical trials and only voluntarily during post-marketing periods (i.e., phase IV clinical trials), but under-reporting is still common during the post-marketing stage [5]. Moreover, as all adverse events cannot be reported during the product development and launch phase due to several inherent limitations of clinical trials, practitioners and regulators must rely on long-term spontaneous reporting by health professionals and patients [6]. Many device manufacturing industries have also started the concept of living data mining techniques to make the regulatory authorities sure that no new device-induced signals arise as with the device use [5].

### Pharmacovigilance and Emergence of Materiovigilance

Pharmacovigilance (Pv) is a science dealing with the continuous identification, assessment, evaluation, and prevention of acute and chronic adverse reactions caused by newly launched and already marketed medicines [5–10]. Pv these days also covers drug therapy-related problems (DTRPs) shown by herbals, traditional, complementary, and alternative medicines (TCAMs), biologicals and blood products, medical devices, and vaccines [6, 11, 12]. Pharmacoepidemiologic studies are popular these days to evaluate the safety and effectiveness of medicines in clinical practice [13]. Materiovigilance (Mv) has similar reporting purposes and approaches as that of Pv but deals with adverse reactions caused by medical devices and their prevention strategies [2–4, 8, 14, 15]. For example, devices may lead to adverse events due to their design or manufacturing problems, inadequate maintenance, irrational storage and logistics, poor user instructions or training leading to incorrect use, off-label or unapproved use, and many other context-specific issues [16]. Thus, like all medicines, all medical devices may also have certain risks. Therefore, monitoring the safety of these devices helps withdraw dangerous or potentially dangerous devices from the market and eliminate defects to improve their quality and performance standards [8, 15].

### Global Materiovigilance Initiatives

The International Medical Device Regulators Forum (IMDRF), comprising 10 countries, such as the USA, Japan, EU, China, South Korea, and India, was set up in 2011 to introduce the concept and implementation of the Mv program to monitor medical devices associated adverse events (MDAEs) and to harmonize international medical device regulation via Mv [8]. The materiovigilance program of India (MvPI), launched on July 6, 2015, helps to systematically collect safety data on device use from Indian population, to monitor MDAEs, to raise awareness among health professionals on their reporting, to monitor benefits versus risks, to generate evidence-based suggestions on safety, and to communicate findings to the stakeholders and regulatory authorities [2, 14, 15, 17]. Table 1 shows Mv initiatives in some countries.

**Table 1:** Materiovigilance regulation in some countries.

Countries	Classification system	Descriptions	Regulatory authority	References
Japan	General (Class I): e.g., X-ray films, in vitro devices Controlled (Class II): e.g., Ultrasound devices, electronic endoscopes Specially Controlled (Class III and IV): Class III items: e.g., Bone prosthesis along with dialyzer Class IV items: e.g., Pacemakers, stent graft	Device notification (for Class I devices), certificate (for Class II) or approval (for class III and IV devices) is essential Clinical trials are unnecessary for class I and II devices but occasionally required for class III and compulsory for class IV devices	Pharmaceuticals medical devices agency (PMDA)	[38]
USA	Class I (lowest risk): e.g., Handheld surgical instruments, tongue depressors, gauze, crutches, elastic bandages, examination gloves, and lancets Class II (intermediate risk): e.g., Cardiac monitors, infusion pumps, surgical drapes, oxygen masks, contact lenses, catheters, syringes, anesthesiology devices, toxicology devices, suture and needles, ultrasound imaging systems, blood glucose meters, and infusion pumps (about 43% of devices) Class III (highest risk): e.g., Pacemakers, heart valves, coronary stents, cochlear implants, defibrillators, implanted prostheses, silicone gel-filled breast implants, and continuous glucose monitors (about 10% of products)	Class I devices do not injure the recipients and their approval can be received within one week of application These are often exempt from the premarket review due to their assured safety profile, but the FDA should be notified before their commercial sale and distribution Class II devices: Only general controls are not adequate to ensure their safety and effectiveness, and it may take 60 to 177 days to get FDA approval Class III devices: life-sustaining/supporting, most invasive, and require stringent testing and monitoring to receive an approval within 243 days after submission	Center for devices and radiological health (CDRH) of the FDA	[38]
EU	Class I: e.g., Gloves, sterile dressing, elastic bandages Class IIa: e.g., Surgical blades, radiotherapy equipment, suction equipment, infusion pumps Class IIb: e.g., Hemodialyzers Class III: e.g., Ventilators, implants, heart valves Class IV: e.g., Pacemakers, drug-eluting cardiac stents, implantable defibrillators	The higher the classification, the greater is the level of scrutiny required	Medical device directive (MDD)	[39, 40]
United Kingdom	Class I: e.g., Dressings Class IIa: e.g., X-ray film Class IIb: e.g., Blood bags, contact lens care products Class III: e.g., Bone cement, cardiac stents	Class I items pose low risk Class IIa devices have low-to-medium risk Class IIb shows medium-to-high risk Class III items have a high-risk profile	Medicines and healthcare products regulatory agency (MHRA)	[30]
Australia	Class I: e.g., Tongue dispensers, surgical retractors, surgical microscopes Class I (supplied sterile): e.g., Sterile surgical gloves Class I (with a measuring device): e.g., Medicine cup Class IIa: e.g., Dental drills, electrical acupuncture Class IIb: e.g., Surgical lasers, infant incubators, external defibrillators Class III: e.g., Prosthetic heart valve, hip prostheses, heparin-coated catheters Class IV: e.g., Pacemakers, artificial heart, intrauterine contraceptive devices	Class I devices present minimum risks, whereas class I-supplied sterile, class I-incorporating a measuring device, and class IIa produce low-to-medium risks Class IIb devices pose medium-to-high risks, whereas class III and active implantable medical devices (AIMD) yield the highest risks	Therapeutic Goods Administration (TGA)	[41]
New Zealand	Class I: e.g., Sterile dressings (non-medicated), reusable surgical instruments Class I (measuring, low risk): e.g., Volumetric urine bag Class IIa: e.g., Hypodermic needles, suction equipment Class IIb: e.g., Ventilators, orthopedic implants Class III: e.g., Drug-eluting cardiac stents	Class I devices are sterile and pose low risk Class IIa devices are medium-to-low-risk devices Class IIb items pose medium-to-high risk Class III items are high-risk items	MedSafe	[42]
Canada	Class I: e.g., Chemical analyzer Class II: e.g., Urine test strips Class III: e.g., Blood glucose self-testing Class IV: e.g., HIV blood analyzer	Risks are gradually greater for the higher level of classification	Therapeutic products division (TPD) of health Canada	[43]
Russia	Class 1, 2a, 2b, and 3	GOST Standard (Federal Agency for Technical Regulation and Metrology) ensures that all medical devices fulfill well-established Russian benchmarks set by the regulatory authority	Roszdravnadzor	[44]
Brazil	Class I: e.g., Simple surgical instruments, tongue depressor Class II: e.g., Digestive catheters, infusion pumps Class III: e.g., Dialyzers, orthopedic implants Class IV: e.g., Coronary stents	Class I items pose low risk Class II devices have low-to-moderate risk Class III items have high-to-moderate risk Class IV devices pose high risk	National health surveillance agency (Anvisa)	[45]
China	Class I: e.g., Ear probes, scalpels, medical dressing, invasive devices, reusable surgical devices Class II: e.g., Disposable umbilical cords, liquid transportation devices Class III: e.g., Disposable venous infusion needles	Class I items have low risk Class II devices pose moderate risk Class III devices show high risk	China food and drug administration (CFDA)	[46]
India	Class A: e.g., Surgical dressing, suture, alcohol swabs, thermometers, nasopharyngeal swabs, tongue depressors, umbilical occlusion device Class B: e.g., Atrioventricular shunt, transcervical endoscope, oximeter catheter, hypodermic needles, suction equipment, hematology reagent kits, disinfectants, intravenous catheter, rectal catheter, fistula adapter Class C: e.g., Uterine balloon therapy device, vein ablation device, intraocular lenses, lung ventilator, bone fixation plate, biliary stents, bone cement Class D: e.g., Coronary stent, cardiac stents, implantable defibrillator, cochlear implants, heart valve, copper T	Class A items have low risk Class B items pose low-to-moderate risk Class C devices pose moderate-to-high risk Class D items have high risk	Central drug standard control organization (CDSCO)	[47, 48]

### Significance of Materiovigilance

Mv emerged as a modified novel branch of Pv to improve patients’ health and safety by reducing the incidence of MDAEs, developing a framework and mechanism for nationwide patient safety monitoring, generating evidence-based data related to medical devices’ safety, analyzing their risk–benefit ratio, and disseminating the information to the stakeholders. Mv also assists the regulatory authorities in making decisions related to medical devices, such as collaborating with national and international agencies on MDAEs reporting and investigation to prevent potential adverse events in future. It helps practitioners diagnose, monitor, manage, or mitigate disease or injury. In addition, Mv allows manufacturers to improve design and efficiency profiles of medical devices [1–3, 14, 18] and to perform life cycle assessments of devices and report adverse events to avoid complications of counterfeit or substandard devices, such as device breakage and malfunction, entry- and exit-site infections, organ perforations or injuries, need for surgery, and even death [19].

## Methods

### Search Strategy

Four electronic databases (i.e., PubMed/MEDLINE, Scopus, Science Direct) were searched for medical devices-related studies published in English until October 31, 2022. The following search strategies were used to explore and extract relevant articles:

- ‟Materiovigilance” OR ‟Pharmacovigilance” OR ‟Adverse event* report*” OR ‟Adverse effect* report*” OR ‟Adverse outcom* report*” AND (Global healthcare” OR ‟Global healthcare system”)
- ‟Medical devices” OR ‟Medical devices associated adverse events” OR “Medical device recall” AND (‟Regulation” OR “Reporting guidelines”)

### Data Synthesis and Analysis

Information related to materiovigilance was extracted from research articles and review papers and interpreted as tabular forms and figures.

### Safety Specifications to be Considered During Materiovigilance Execution

The safety specifications related to devices can be divided into three risk categories like in case of medicines—identified risks (i.e., prominent and relevant evidence-based adverse events), potential risks (i.e., prominent or suspicious adverse events but without sufficient clinical data), and missing information (i.e., critical for estimating post-marketing safety but not available during risk management plan (RMP) phase) [20]. Some risks caused by devices may be life-threatening to patients, users, and even health professionals due to their interactions with other substances, contraindications, malfunctions, falsifications, technical defects, and compromised efficacy [14]. Hence, the USA, European Union, Japan, and Canada agreed upon the joint medical device regulatory landscape to ensure that safe, effective, and innovative devices are used in health care like in diabetes care in these countries [21].

### Medical Devices Associated Adverse Events Reporting

Important concerns to be addressed in the case of devices are the global consistency in their classification and approval processes, if not possible, at least transparency [22]. All MDAEs such as critical or non-critical, known or unknown, those related to inadequate or incomplete specifications, and frequent or rare events should be reported [5, 14, 15] and evaluated case by case and on an aggregate level [5]. The descriptions of devices and their hazards or suspected risks with previous usage experience can also be reported [15]. Table 2 depicts the information required for the reporting of MDAEs.

**Table 2:** Information required for the reporting of MDAEs [3, 16, 18].

Type/category of information	Details
General information	Date and type of report (initial/follow-up/final/trend)
Reporter details	Type of reporter (manufacturer/importer/health professionals/users/others) with their contact information
Device category	Device type (therapeutic/diagnostic/preventive/others), invasive/non-invasive, single use/reusable, sterile/non-sterile
Device details	Device name, manufacturer’s name, address and contact information, supplier or purchaser, license number, specification, batch/lot/model, quantity of device used, manufacturing and expiry date, period of validity (i.e., shelf life), date of sale
Event description	Event date, implant date, serious/non-serious, description of the event, device operator
Patient information	Patient initial, age, gender, weight and BMI, relevant medical history, and patient outcomes (recovered/not recovered)
Causality assessment	Investigation or action taken, root cause analysis (RCA) of problem, challenge, and dechallenge-related information
Manufacturer’s investigation and action taken	Devise risk analysis report, corrective/preventive action taken, device history review

The adverse events reporting system serves as an essential tool whereby all types of MDAEs can be reported to protect users from unforeseen or unexpected effects and to improve their health and safety. The Global Harmonization Task Force (GHTF) provides the manufacturers’ guidance in reporting MDAEs and information on handling the same and in deciding whether an event is reportable. In the USA, the FDA requires that manufacturers and importers report serious adverse events (SAEs) or malfunctions of medical devices [14].

### Reporting of Medical Devices Associated Adverse Events in Some Countries

The FDA requires that MDAEs must be reported by their manufacturers and importers, including quality problems, defects, and performance errors. Similarly, patients, users, and health professionals can voluntarily report the same in case of treatment failures to improve patient safety [21]. Similarly, manufacturers report the MDAEs to the National Competent Authority (NCA) in Europe within two days, death reports and other deteriorating health problems within ten days, and other non-serious incidents within 30 days. In contrast, health professionals report these to the NCA and the manufacturers [23]. Health Canada reviews applications of new and remodeled or redesigned devices in Canada and requires that deformities be reported by the manufacturers and the consumers on their respective forms, depending on the device category. A potential death case should be reported to Health Canada within ten days, whereas a non-critical case should be within 30 days [24].

**Figure 1:**
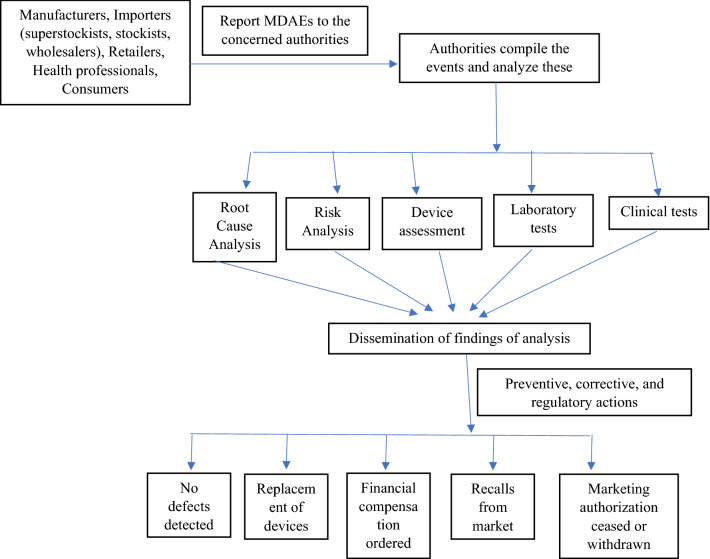
Brief schematic representation of medical devices-related reporting. *Concept developed based on the research of Tase et al. (2021) [37].

Manufacturers and sponsors report per the guidelines set by the Therapeutic Goods Act (TGA) in Australia, and TGA maintains records containing a detailed history of all lots, components, and potential health hazards for a maximum of five years [25]. The MDAEs are reported by the marketing authorization holder (MAH) within two weeks, and any death events or critical cases are reported within 30 days in Japan [26]. Likewise, China Food and Drug Administration (CFDA)’s National Center for ADR Monitoring collects adverse event reports and manages post-approval surveillance in China, even at regional and provincial levels. Injury-related events should be reported to the local ADR monitoring centers within 15 days and death events to the National Center. However, manufacturers and distributors can also directly submit the reports to the National Center in the country [27]. In India, health professionals, manufacturers or importers, and consumers can voluntarily report MDAEs to date. However, manufacturers and importers should mandatorily report any serious events to the Central Drugs Standard Control Organization (CDSCO), which then takes the necessary regulatory decisions on the safety of devices as per the established procedures of MvPI [3, 17]. Figure 1 gives the reporting framework for devices-related concerns, and Table 3 details reporting guidelines for MDAEs in some countries.

**Table 3:** Reporting guidelines for MDAEs in some countries.

	USA	Canada	EU	Australia	Japan	China	India
Regulatory authority	USFDA	Health Canada	European medicines agency (EMA)	TGA	Ministry of health, labor and welfare (MHLW)	China FDA (CFDA)	CDSCO
Guidelines followed	21CFR803A	Canadian medical devices regulations, section 60 and 61	Adverse Incident Tracking System (AITS)	Australian Medical Devices Guidelines—guidance document number 11, version 1.7	Pharmaceutical affairs law Article 77–4-2	Provisions for medical device adverse event monitoring and re-evaluation	Guidance document MvPI version 1.2
Device tracking	Post-marketing surveillance (PMS)	Classified by the medical devices bureau (MDB) according to the Canadian Risk-Based Classification System (RBCS)	Users report device-related incidents, which are later allocated reference numbers for investigation purposes	Done during PMS	Reporting of Fuguai Reports (AEs) to the MHLW	Any organization/ individual can report an adverse or suspected adverse event to the CFDA	
Adverse event reporting	Serious injury/ death is to be reported to the FDA User facilities (e.g., hospital, nursing home, and outpatient diagnostic facilities) should report serious injuries and deaths to both FDA and manufacturer	A trend report of adverse events should be done	Reporting of risks identified during PMS	Reports of medical device vigilance (MDV) are submitted to TGA	MHLW delegates the report reviewing task to the Pharmaceutical and Medical Device Agency (PMDA)	The CFDA collects, analyzes, evaluates, and controls adverse events-related data	Anyone can report MDAEs to the CDSCO
Reporting timeframes	30 days for the manufacturers to report the death, serious injuries, and malfunctions, and five days to report an event requiring remedial action to prevent risks Ten days to report death and serious injuries by user facilities	Ten days to report any serious event or death by the manufacturer or importer 30 days to report similar events by the patients, users or other people	Two days to report serious public health threats Ten days to report serious injury or death 30 days to file reports by the user facilities	- Within two days for significant public health threats or concerns - Within ten days for a serious injury or death - Within 30 days for a near event	15 days for serious adverse events	Reporting of serious adverse events (SAEs) within 15 days of occurrence- death within five days, and normal MDAEs within 30 days	Any SAE like deaths, serious injuries, malfunction, etc. to be reported within 15 days of identification Non-serious events reporting to be done within 30 days of occurrence
Reporting mechanism	Form 3500A Form 3419 for user facilities	Mandatory Medical Device Problem Reporting Form for Industry	Reporting and recording system for incidents and corrective actions	Submitted using the Medical Device Incident Reporting (MDIR) System	Reports compiled in the PMDA Safety Department database	National Medical Device Adverse Event Monitoring Platform	Reporting forms developed by MvPI are duly signed and sent to the nearest medical device monitoring center (MDMC) or directly to the National Collaborating Center
Penalties to the violators	Product recall, seize and import refusal by the FDA if the Federal Food, Drug and Cosmetic Act (FFDCA) is violated Filing a case to the court, and the court can solicit criminal penalties such as prison sentences depending on the case	Manufacturer, importer or distributor warned to recall or correct the problems	Massive penalty amounts and imprisonments on repetition or serious first-time law violations in Germany, France, and UK	Fines of up to USD 15,000 for manufacturer or suppliers’ firms	Warned about recalls or changes in labeling or package inserts	Device permit is rejected, and no application for the related license or permit acceptance within five years	Five lakhs rupees fine or one-year imprisonment or both for the manufacturer, importer, and seller
References	[38]	[24, 49]	[23]	[25, 50]	[26, 51]	[27]	[3, 17]

*CDSCO* central drugs standard control organization (CDSCO), *FDA* food and drug administration, *TGA* therapeutic goods administration, *USFDA* United States food and drug administration

### Reporting Framework for Medical Devices-Related Issues

A simple and brief schematic representation of medical devices-related reporting can be explained below:

### Not-Reportable Incidents

As every adverse event cannot necessarily be documented, some regulatory authorities define not-reportable events as adverse events caused by patients’ pre-existing health conditions. Similarly, events cannot be reportable if the shelf life expires before its use by the patients. Also, if the manufacturers mentioned the expected side effects in their product labeling, these cannot be considered reportable events. Above all, if the patients’ abnormal use pattern of behavior induced any untoward events, these can also not be reportable [14].

### Social Media and Their Role in Materiovigilance

Social media, like LinkedIn, Facebook, Twitter, YouTube, help to provide users with rapid and up-to-date information on rational selection and undesired events and promote health- and science-related developments and issues [28, 29]. They also contribute to sharing information related to medical device recalls if any defects are reported. This will ultimately make the users and practitioners aware of the latest regulatory actions or provisions related to the device. However, over-reliance on information disseminated on social media platforms for every piece of information may create havoc for users, especially physical hazards, and mental distress. Hence, critical analysis of information shared on social media with its source verification is essential to avoid potential misinformation because misinformation may be even worse than no information [28].

### Recall of Devices

Devices are recalled for multiple reasons, some of which are defects in the products or their potential impacts on morbidity and mortality to the users [2]. The guidelines of Medicine and Healthcare Product Regulatory (MHRA) and GHTF stated that devices could be returned to their manufacturers or vendors as primary methods. Also, these can be alternatively subject to modification, remodeling, substituting of newer ones, or dismantling as part of a regular device recall [30].

Regulatory authorities like the FDA or device manufacturers can recall the products when deemed to pose any health complications to the patients. Whenever the manufacturers even voluntarily recall the products, they should notify the FDA immediately. The FDA may recall devices based on their categories or severity of harm and may update its website with reasons of such recalls [31]. Class I recalls apply to the highest risks to patients, Class II recalls refer to those with a moderate risk, whereas Class III recalls meaning those with low-risk profiles [32]. Since the recall list is quite comprehensive, a brief account of some medical devices recalled from the market within the last ten years has been presented in Table 4.

**Table 4:** Medical devices recalled from the market within the last 10 years.

Date of recall	Country	Medical device	Reason of recall	References
January, 2022	USA	Sevoflurane vaporizer, Maquet Filling for Flow Family Anesthesia Systems	Chemical breakdown of sevoflurane resulted in inhalation or skin exposure to harmful chemicals, which ultimately irritated the respiratory tract and caused edema of lungs, and hypocalcemia	[52]
October, 2021	USA	Ellume COVID-19 home test	False-positive test results	[31]
July, 2020	India	Coronavirus testing kits	Non-performance reports from Punjab, Rajasthan, and Karnataka	[8]
August, 2020	USA	Alaris system pump module and pump module door assembly replacement kits	One or more unresponsive keys, leading to delay in infusion and increased risk of harm and even death	[53]
2020	Japan	Abenomask	Complaints about stains, insects, and mold	[8]
October, 2019	USA	Medfusion® syringe pumps	Therapy-related malfunctioning alarms related to battery	[31]
July, 2019	USA	Allergan breast implant	High risk of anaphylactic large cell lymphoma	[54]
January, 2019	USA	Vial2Bag fluid transfer systems of the West Pharmaceutical Services	Risk of faulty transfer of medicine from vial to bag leading to overdose or underdose of medication	[55]
April, 2017	USA	Zimmer Biomet spinal fusion stimulators	Presence of harmful or toxic chemicals	[8]
2010	India	ASR XL acetabular hip replacement system	Repeat surgery due to the release of metallic debris from metal implants into the bloodstream	[8]

### Role of Health Professionals in Reporting Device-Related Issues

Health professionals such as surgeons, physicians, nurses, and pharmacists can report medical device-related concerns by building and enhancing individual and institutional capacity to report and tackle the device-related adverse effects. This can be achieved with the database information system that generates signals for medical devices. They can also educate and train colleagues and patients to raise their awareness of the importance of Mv in device recalls if a fault should appear in practice since the main thing is the initiation and development of an institutional culture for reporting MDAEs for their future prevention [33].

### Role of Consumers in Reporting Device Failures

Consumers in the USA are somewhat aware of reporting MDAEs like quality issues and administration errors experienced with devices and filling the Form FDA 3500B and reporting via MedWatch voluntary reporting system [34]. In Australia, the Therapeutic Goods Administration (TGA) has developed a website to empower the users’ reporting practice [35]. Similarly, in New Zealand, the MedSafe system developed a form for the consumers to be filled in the Word document format and send via email on its website [36]. The reporting by the consumers helps practitioners, pharmacists, and policymakers to know the users’ perspectives and concerns on devices in strengthening the reporting systems and ultimately in focusing on the quality products.

### Challenges and Solutions Associated with Materiovigilance

As in the case of Pv programs, Mv also faces similar challenges to getting implemented up to the practitioners’ and consumers’ level. The consumers and practitioners generally perceive reporting as tedious and lack awareness on the significance and procedures of reporting (e.g., what, how, where and when to report the incidents). In addition, the lack of stringent regulatory mechanisms to make the reporting mandatory makes its implementation challenging in real practice settings. The practitioners’ negative perception of MDAEs reporting as if these are being directed at reporting their mistakes has also become a significant barrier. Moreover, a lack of trained reporters and conducive facilities are also hindering the successful implementation of Mv [37]. Regulatory authority’s initiation in affixing the mandatory package inserts and summary product characteristics (SPC) of the devices may help tackle some hindrances in implementing Mv.

## Conclusion

Developed countries like the UK, the USA, New Zealand, Australia, and some developing countries like China and India have developed materiovigilance (Mv) mechanisms in reporting medical device-associated adverse events (MDAEs). Still, many developing countries lack such manufacturing facilities, rely on imports, and lack stringent Mv programs. Hence, policymakers must develop Mv programs to empower health professionals and consumers to report the MDAEs to prevent a recurrence. The package insert of medical devices must include a section highlighting the potential risks with their prevalence pattern. While the pharmacovigilance programs globally are taking good shape, much more must be done to initiate and strengthen Mv programs, at least in the countries reviewed in the present study but preferably globally.

## Data Availability

All data supporting the findings of this study are contained within the manuscript. Any additional information regarding the study including the questionnaires would be shared by the corresponding author upon request.
